# A novel patient-derived orthotopic xenograft model of esophageal adenocarcinoma provides a platform for translational discoveries

**DOI:** 10.1242/dmm.041004

**Published:** 2019-12-17

**Authors:** Omkara Lakshmi Veeranki, Zhimin Tong, Alicia Mejia, Anuj Verma, Riham Katkhuda, Roland Bassett, Tae-Beom Kim, Jing Wang, Wenhua Lang, Barbara Mino, Luisa Solis, Charles Kingsley, William Norton, Ramesh Tailor, Ji Yuan Wu, Sunil Krishnan, Steven H. Lin, Mariela Blum, Wayne Hofstetter, Jaffer Ajani, Scott Kopetz, Dipen Maru

**Affiliations:** 1 Department of Pathology, The University of Texas MD Anderson Cancer Center, Houston, TX 77030, USA; 2 Department of Translational Molecular Pathology, The University of Texas MD Anderson Cancer Center, Houston, TX 77030, USA; 3 Department of Biostatistics, The University of Texas MD Anderson Cancer Center, Houston, TX 77030, USA; 4 Department of Bioinformatics and Computational Biology, The University of Texas MD Anderson Cancer Center, Houston, TX 77030, USA; 5 Department of Imaging Physics, The University of Texas MD Anderson Cancer Center, Houston, TX 77030, USA; 6 Department of Veterinary Medicine and Surgery, The University of Texas MD Anderson Cancer Center, Houston, TX 77030, USA; 7 Department of Radiation Physics, The University of Texas MD Anderson Cancer Center, Houston, TX 77030, USA; 8 Department of Gastrointestinal Medical Oncology, The University of Texas MD Anderson Cancer Center, Houston, TX 77030, USA; 9 Department of Radiation Oncology, The University of Texas MD Anderson Cancer Center, Houston, TX 77030, USA; 10 Department of Thoracic and Cardiovascular Surgery, The University of Texas MD Anderson Cancer Center, Houston, TX 77030, USA

**Keywords:** Patient-derived orthotopic xenograft mouse model, Esophageal adenocarcinoma, Gastroesophageal junction cancer, Mouse models, Microenvironment

## Abstract

Mouse models of gastroesophageal junction (GEJ) cancer strive to recapitulate the intratumoral heterogeneity and cellular crosstalk within patient tumors to improve clinical translation. GEJ cancers remain a therapeutic challenge due to the lack of a reliable mouse model for preclinical drug testing. In this study, a novel patient-derived orthotopic xenograft (PDOX) was established from GEJ cancer via transabdominal surgical implantation. Patient tumor was compared to subcutaneously implanted patient-derived tumor xenograft (PDX) and PDOX by Hematoxylin and Eosin staining, immunohistochemistry and next-generation sequencing. Treatment efficacy studies of radiotherapy were performed. We observed that mechanical abrasion of mouse GEJ prior to surgical implantation of a patient-derived tumor *in situ* promotes tumor engraftment (100%, *n*=6). Complete PDOX engraftment was observed with rapid intra- and extraluminal tumor growth, as evidenced by magnetic resonance imaging. PDOXs contain fibroblasts, tumor-associated macrophages, immune and inflammatory cells, vascular and lymphatic vessels. Stromal hallmarks of aggressive GEJ cancers are recapitulated in a GEJ PDOX mouse model. PDOXs demonstrate tumor invasion into vasculature and perineural space. Next-generation sequencing revealed loss of heterozygosity with very high allelic frequency in *NOTCH3*, *TGFB1*, *EZH2* and *KMT2C* in the patient tumor, the subcutaneous PDX and the PDOX. Immunohistochemical analysis of Her2/neu (also known as ERBB2), p53 (also known as TP53) and p16 (also known as CDKN2A) in PDX and PDOX revealed maintenance of expression of proteins found in patient tumors, but membranous EGFR overexpression in patient tumor cells was absent in both xenografts. Targeted radiotherapy in this model suggested a decrease in size by 61% according to Response Evaluation Criteria in Solid Tumors (RECIST), indicating a partial response to radiation therapy. Our GEJ PDOX model exhibits remarkable fidelity to human disease and captures the precise tissue microenvironment present within the local GEJ architecture, providing a novel tool for translating findings from studies on human GEJ cancer. This model can be applied to study metastatic progression and to develop novel therapeutic approaches for the treatment of GEJ cancer.

This article has an associated First Person interview with the first author of the paper.

## Abstract (precis)

**Editor's choice:** The patient-derived orthotopic xenograft model of gastroesophageal adenocarcinoma maintains the morphological and molecular characteristics despite being highly heterogeneous, emphasizing its use as a powerful investigational platform to evaluate new therapies.

## INTRODUCTION

Since the 1970s, gastroesophageal (GE) cancers are an increasing cause of cancer burden globally (Blot et al., 1991; Fitzmaurice et al., 2015). Despite recent treatment advances, including the use of neoadjuvant chemoradiation therapy (Mokdad et al., 2018), localized esophageal and/or gastroesophageal junction (GEJ) adenocarcinoma remains a highly aggressive cancer with 5-year survival rates of less than 30% (Fitzmaurice et al., 2015). One factor limiting improvements in treatment efficacy is a lack of preclinical models that recapitulate the growth pattern and radiological and pathological characteristics of patient tumors and that can be used to assess response to chemoradiation. Conventional preclinical models do not entirely recapitulate the human disease and hamper development of new treatments. Cell line xenografts implanted subcutaneously or orthotopically lack the architectural complexity of patient tumors, which include intratumoral inflammatory cells, blood vessels and the presence of stromal elements (Becher and Holland, 2006). Thus, genetically engineered mouse models (GEMMs) (Becher and Holland, 2006) and patient-derived xenografts (PDXs) have emerged as promising translational platforms due to their resemblance to human tumors (Hidalgo et al., 2014; Siolas and Hannon, 2013). Well-established GEMMs of GEJ cancers (HK-ATPase hIL-1β, IL-1β;NFκB^EGFP^, IL-1β;Rag2^−/−^, Villin-cre,Smad4^F/F^,Trp53^F/F^, Cdh1^F/wt^ and Pdx1-cre, Trp53^F/F^, Cdh1^F/F^) (Park et al., 2015; Tu et al., 2008) have contributed significantly to our understanding of GEJ tumor biology. Owing to the histological differences between mouse and human GEJ, GEMMs for GE cancers have been extremely difficult to develop (Tétreault, 2015). In the human GEJ, the nonkeratinizing esophageal squamous epithelium is formed between the distal esophagus, composed of squamous mucosa, abruptly transitioning to gastric columnar mucosa. In mouse, this squamocolumnar junction is keratinized and is within the stomach (Treuting et al., 2011). The keratinized epithelium in mouse GEJ epithelium renders the mice resistant to injuries and to the development of GE cancers. Moreover, transgenic mice such as p63^−/−^ have limitations as a robust GEJ cancer model as they do not survive to adulthood (Wang et al., 2011). Although immunocompetent murine cancer models and GEMMs develop spontaneous *de novo* tumors with a co-evolving microenvironment and an intact immune system, these systems are biased towards mouse-specific efficacy.

PDX models have become invaluable in translational cancer research because they retain a patient's tumor characteristics and tumor heterogeneity, and predict sensitivity to treatment better than cell-line xenograft models. They preserve tumor heterogeneity of parental tumors at histological and molecular levels even after multiple passages in mice (Choi et al., 2016; Dodbiba et al., 2015). PDXs are valuable tools for personalized medicine despite human stroma being replaced by murine stroma. PDXs at subcutaneous and other heterotopic sites are attractive because of their technical simplicity. However, the subcutaneous sites differ from the tubular gastrointestinal tract in terms of anatomical boundaries and the tumor microenvironment, limiting the applicability of subcutaneous PDX models for understanding tumor growth and response to treatment. Further, aggressive GEJ cancers are characterized by rich vasculature, lymphatic vessels, increased tumor-associated macrophages (TAMs) and cancer-associated fibroblasts. Subcutaneous PDX limits the usefulness of the PDX model in metastatic, angiogenic and tumor microenvironment studies. To overcome this limitation, a GEJ patient-derived orthotopic xenograft (PDOX) was established by directly implanting the GE cancer at the mouse GEJ, which enabled tumor growth similar to that of human tumors.

## RESULTS

### Establishing a PDOX mouse model of GEJ cancer

**Fig. 1.**
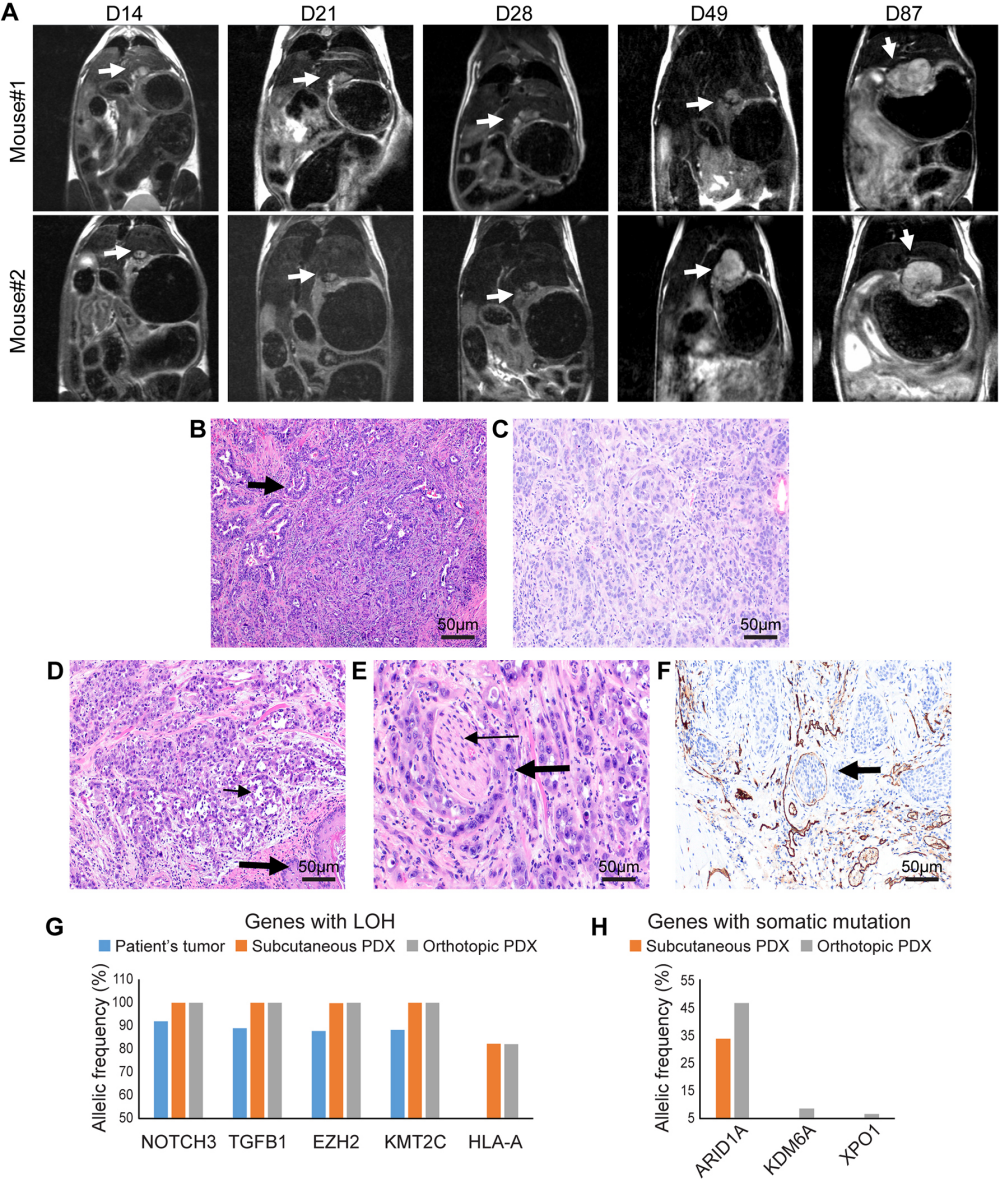
**The PDOX mouse model of esophageal adenocarcinoma mimics the tumor growth pattern, and histopathological and molecular characteristics of the patient tumor.** (A) MRI (coronal view) demonstrating growth of esophageal/GEJ PDOX (arrows) in two mice at day (D) 14, D21, D28, D49 and D87. In mouse #1, at D49, there is clear evidence of intra- and extraluminal spread in the esophageal wall. (B-F) Histopathological evaluation by Hematoxylin and Eosin (H&E) staining and immunohistochemistry at 200× magnification: (B) glandular architecture (arrow) and cytologic features of the patient's tumor; (C) glandular architecture and cytologic features of subcutaneously implanted PDX; (D) PDOX exhibiting glands and cellular features (thin arrow) similar to the histologic features of the patient tumor (squamous epithelium of esophagus, thick arrow); (E) a focus of perineural invasion in PDOX (nerve bundle, thin arrow; perineural tumor, thick arrow); (F) immunohistochemistry of CD31 depicting lymphovascular infiltration (arrow) in GEJ PDOX. (G,H) Next-generation sequencing results demonstrating allelic frequency (%) for genes with loss of heterozygosity (LOH) (G) and somatic mutation (H).

PDOX models are rapidly gaining popularity, as they have a long history of mimicking patient tumor growth and metastasis in cancers of the colon, pancreas, ovary, lung and stomach (Furukawa et al., 1993b; Hoffman, 2015). Unlike other sites in the tubular gastrointestinal tract (Furukawa et al., 1993a,b), no PDOX models are available for esophageal and/or GEJ cancers. Therefore, we established an esophageal/GEJ PDOX model by transabdominally implanting a biopsy sample of esophageal adenocarcinoma at the distal esophagus/GEJ of six female SCID mice (Fig. S1A-I). Magnetic resonance imaging (MRI) with and without a gadolinium-based contrast agent (Magnevist) was used to visualize tumor boundaries and characterize tumor growth. T1-weighted images with contrast clearly showed enhancement of the tumor boundaries (Fig. S1J,K) and regions of hyperintensity and hypointensity, indicating a mixture of viable and necrotic tumor tissue. MRI studies conducted 7 days after surgical implantation demonstrated 100% (*n*=6) engraftment (Fig. S1I). MRI also showed an increase in the primary tumor size at each evaluated time point, indicating that the GEJ PDOXs grew over time (Fig. 1A). The mean tumor volume (±s.e.m.) was 5.2±1.1 mm^3^ at Day 14 after implantation, 4.3±1.2 mm^3^ at Day 21, 21.8±10.9 mm^3^ at Day 28, 18.6±12.3 mm^3^ at Day 49 and 84.5±4.6 mm^3^ at Day 87.

### The PDOX mouse model mimics the patient GEJ tumor at histological and molecular level

The histopathological features of the PDOX tumors were very similar to those of the patient tumor used to generate the PDOX (Fig. 1B-F). The PDOXs exhibited small glandular and single-cell architecture with intracellular mucin (Fig. 1D), as did the patient tumor (Fig. 1B). An invasive growth pattern with transmural extension to the mucosa and mucosal ulceration (Fig. 1D) resembled the pattern observed on esophagogastroduodenoscopy in the patient. The tumor stroma was characterized by desmoplastic changes and mononuclear inflammatory infiltrates. Immunohistochemical analysis using an antibody specific to the CD68^+^ human epitope depicted TAMs in patient tumor alone and not in subcutaneous PDX and PDOX (Fig. S2). Clinically important tumor microenvironment hallmarks of aggressive behavior such as perineural (Fig. 1E) and intravascular invasion (Fig. 1F) were also observed. Lymphovascular infiltration in the GEJ PDOX was depicted by CD31 (also known as PECAM1) immunohistochemistry (Fig. 1F).

Loss of heterozygosity (LOH) with very high allelic frequency was observed in *NOTCH3*, *TGFB1*, *EZH2* and *KMT2C* in the patient tumor, the subcutaneous PDX and the PDOX. An additional LOH event was observed in major histocompatibility complex, class IA (*HLA-A*) in the subcutaneous PDX and PDOX, but not in the patient tumor (Fig. 1G; Table S1), raising the possibility of the evolution of a tumor cell clone(s) with expression of neoantigens in xenografts. The PDOX and subcutaneous PDX exhibited somatic single-nucleotide variants (SNVs) in *ARID1A* (in-frame loss of codon, allelic frequency 34% and 47%) that were not observed in the patient tumor. Additional low-frequency SNVs in *KDM6A* (in-frame gain of codon, allelic frequency 7%) and *XPO1* (splice region variant, allelic frequency 6%) were identified in the PDOX, but not in the patient tumor or the subcutaneous PDX (Fig. 1H; Table S2). The frequency of SNVs and LOH found in the PDOX but not in the patient tumor was consistent with expectations for subsequent generations of a tumor.

**Fig. 2.**
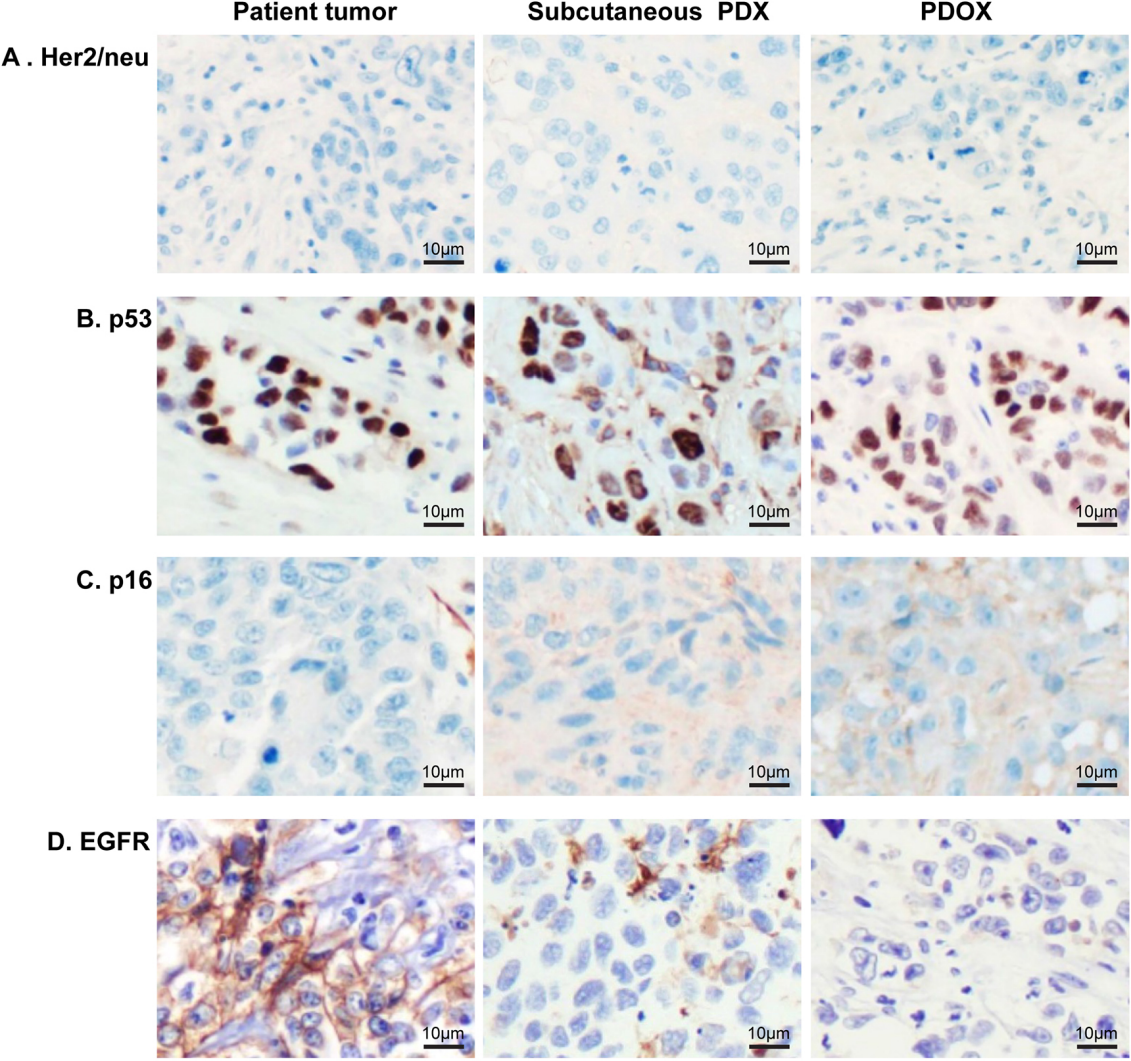
**Immunohistochemical characterization of the PDOX mouse model of esophageal adenocarcinoma compared to patient tumor.** (A) Immunohistochemistry staining for Her2/neu was negative in all tumor cells. (B) p53 demonstrated nuclear staining across all tumor cells. (C) Tumor cells were negative for p16 staining with weak to moderate stromal staining in PDX and PDOX. (D) EGFR membranous staining present in patient tumor cells was lost in PDX and PDOX. Magnification, ×200.

Tumor cells in parental tumor, subcutaneous PDX and PDOX had a similar pattern of staining for Her2/neu (also known as ERBB2), p53 (also known as TP53) and p16 (also known as CDKN2A) by immunohistochemistry (Fig. 2 and Table 1). Her2/neu expression was absent (score 0 of 3) in parental tumor, subcutaneous PDX and PDOX (Fig. 2A). Nuclear p53 staining was observed in 60% or more tumor cells in the parental tumor and was retained in the subcutaneous PDX and PDOX (Fig. 2B). Nuclear or cytoplasmic p16 in the tumor cells was not seen in the patient tumor or in subcutaneous PDX and PDOX (Fig. 2C). Interestingly, membranous staining for EGFR was observed in more than 95% of the tumor cells of the patient tumor, whereas staining was absent in all the cells of subcutaneous PDX and PDOX (Fig. 2D).

**Table 1.**
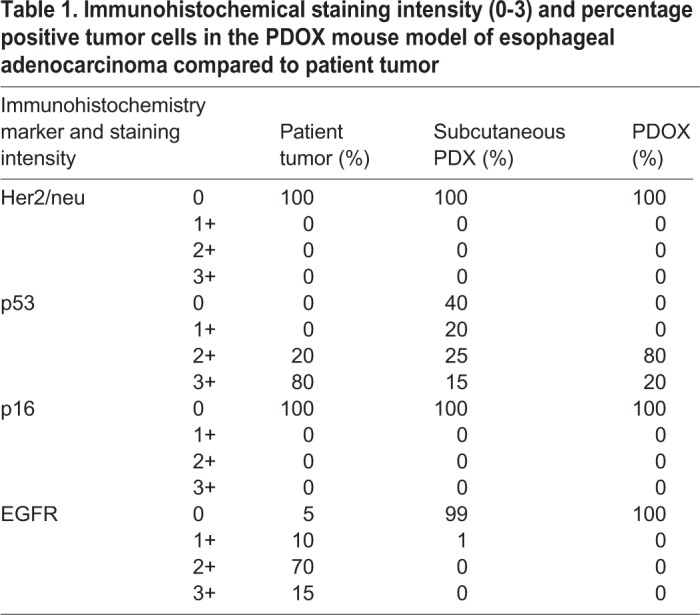
**Immunohistochemical staining intensity (0-3) and percentage positive tumor cells in the PDOX mouse model of esophageal adenocarcinoma compared to patient tumor**

### The PDOX mouse model mimics the GEJ tumor growth pattern in patient tumor

**Fig. 3.**
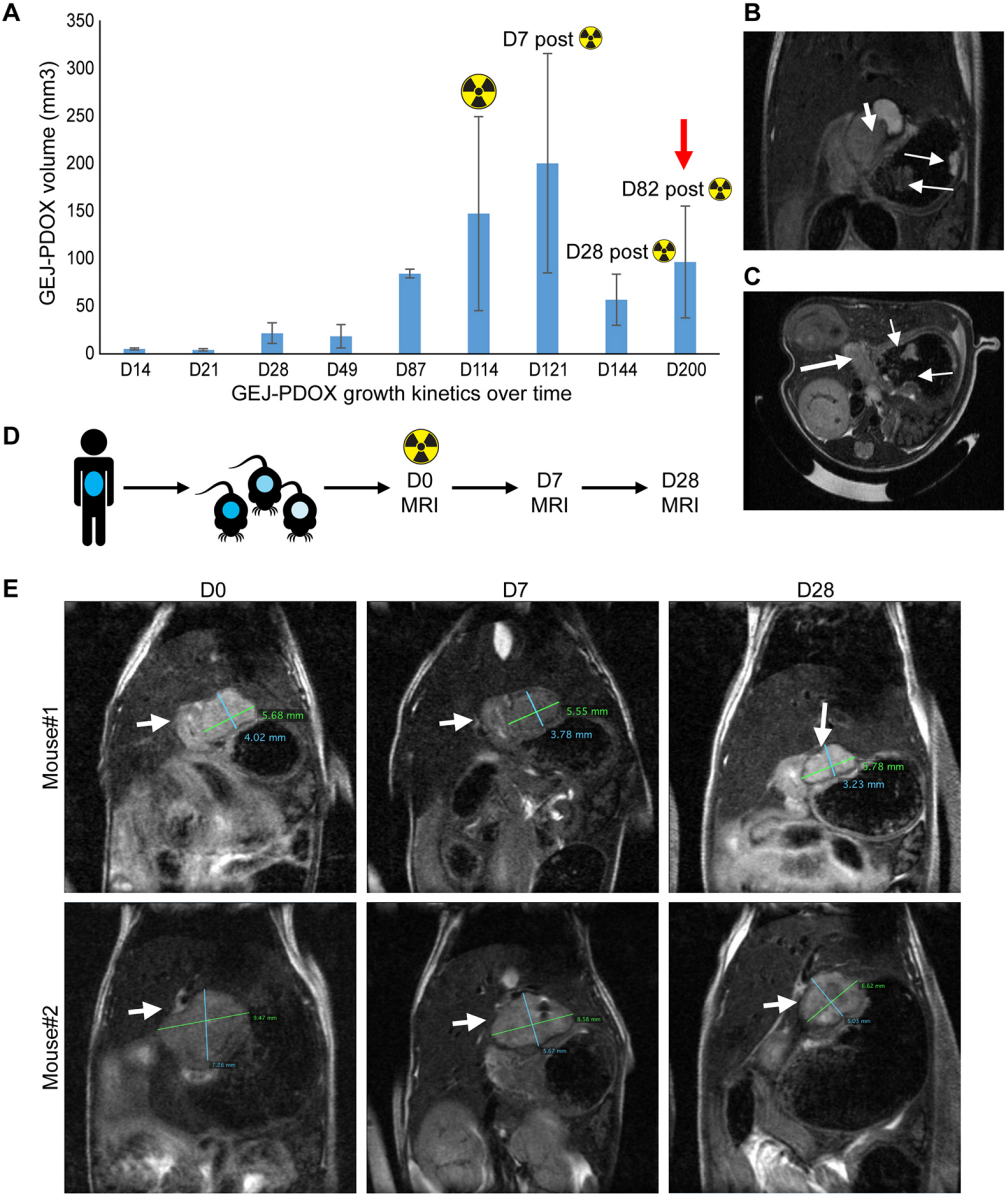
**Growth kinetics and response to targeted radiotherapy in the esophageal adenocarcinoma/GEJ PDOX mouse model.** (A) Growth rate of PDOX over time. The PDOXs were irradiated at D114 after implantation using a 250-kVp x-ray beam at a dose of 10 Gy. At D82 after radiation, multifocal lesions were found within the esophageal and gastric wall (red arrow). (B,C) MRI images showing discontinuous spread of GEJ PDOX within the esophageal and gastric wall on coronal (B) and axial (C) view. The thick arrows indicate the primary PDOX; the thin arrows indicate discontinuous lesions. (D) Treatment algorithm for radiation studies. The PDOXs were irradiated (10 Gy) on D0. (E) Evaluation of response to radiotherapy by MRI at D0, D7 and D28 (arrows indicate the PDOX).

Tumor growth kinetic studies revealed continuous tumor spread (Figs 1A and 3A) to the intraluminal and extraluminal esophageal wall and stomach wall and discontinuous spread in the periesophageal and perigastric soft tissue, mimicking the local growth patterns of esophageal adenocarcinoma in patients. Discontinuous multifocal lesions were observed within the lining of the gastric wall at Day 200 after implantation (i.e. Day 82 after irradiation), suggesting persistent growth of the tumor (Fig. 3B,C).

### The PDOX mouse model recapitulates response to radiation in GEJ cancer patients

To determine how well the PDOX model recapitulated the effects of radiation treatment in patients, we administered a radiation dose of 10 Gy to the PDOX tumors. The radiation treatment field (Fig. 3D; Fig. S3) was comparable to that in a typical treatment plan delivered to patients. The radiation dose was targeted at the tumor in the distal esophagus and GEJ using a 5-mm-thick Wood's metal (Cerrobend**)** block with a 2.0 cm×1.5 cm window placed directly above the GEJ (Fig. S3). Both the Response Evaluation Criteria in Solid Tumors (RECIST) and tumor volume measurements showed a decrease in the tumor burden in the PDOX model 28 days after radiation treatment (Fig. 3E). Radiation-treated tumors decreased in size by 61% according to RECIST, indicating a partial response to radiation therapy. At necropsy, analysis of tumor volume measurements showed a decrease of 49% (68.3±26.9 mm^3^) at 30 days after irradiation. The gross pathological volume of the esophageal/GEJ PDOX tumors was linearly correlated with both the volumetric measurements (r=0.9, *P*<0.001) and RECIST (r=0.9, *P*<0.001). On autopsy, no significant radiotoxicity was noted in the GEJ, the surrounding soft tissues, lung and liver by macroscopic and histopathological examination.

## DISCUSSION

In summary, we generated a novel orthotopic mouse model of esophageal/GEJ adenocarcinoma and demonstrated effective delivery of radiation to this PDOX model. The orthotopic mouse model of esophageal/GEJ adenocarcinoma mimics the bioavailability of radiation and likely chemotherapy or targeted therapy in patients (Malaney et al., 2014; Stewart et al., 2015; Zhang et al., 2013). The PDOX models are more suitable for co-clinical trials as they represent a next-generation approach to optimizing oncology drug development by a more profound and timely understanding of tumor response to experimental agents, while helping to advance clinical development plans. Given the similarities in the imaging and pathologic characteristics of this PDOX model and the patient's tumor in the esophagus/GEJ, in addition to its utility in metastatic, angiogenic and tumor microenvironment studies, we conclude that this model is better than subcutaneous PDX models for studying tumor biology. Expression of CD68 (a pan-macrophage marker specific for human antigen) was found to be positive only in the patient tumor stroma and not in the subcutaneous PDX and PDOX, suggesting that the TAMs in PDX and PDOX may have been replaced by mouse macrophages. Loss of EGFR overexpression on tumor cells observed in subcutaneous PDX and PDOX indicated EGFR inactivation in the PDX model for this particular tumor. Previous reports suggest that EGFR amplification seen in patient tumors is often lost in cell culture but not in xenografts developed from patient tumors (Pandita et al., 2004; Stockhausen et al., 2011). Since the primary antibody used to stain EGFR was human specific, it is unlikely that absence of EGFR in tumor cells in xenografts is due to technical issues or antibody clone. Therefore, the possibility of a new tumor cell clone with loss of EGFR expression in xenografts cannot be excluded. This finding needs to be tested in a larger sample size to determine the presence and significance of EGFR loss in PDXs compared to the parental tumor. Although our model did not demonstrate distant metastasis, metastasis developing from a PDOX model like this one would be more representative of a stage-IV esophageal adenocarcinoma than would any other available models. Further, no significant radiotoxicities, such as esophagitis, gastritis, pneumonitis and inflammation, in liver adjacent to the targeted tumor mass indicate that the GEJ PDOX model has applications in radiotherapy-based studies for concurrent examination of tumor responses to radiation and radiation damage to normal tissues. The limitations of this study include few tumor samples from two patients, as only a portion of PDX can be established. Tumors from both patients were engrafted without any complications from the surgical implantation. Further optimization and expansion of this model from multiple patients for understanding metastatic spread, circulating tumor markers and the efficacy of novel therapeutic agents is warranted. The orthotopic mouse model of esophageal/GEJ adenocarcinoma makes it possible to test multiple therapeutic options for prioritizing patient treatment, which is not possible to carry out in a clinical setting. Despite inherent challenges, PDOX systems are likely to become increasingly useful tools as they closely recapitulate the heterogeneity of human tumors and could facilitate more-efficient oncology drug development.

## MATERIALS AND METHODS

Fresh tissue samples from surgically excised cutaneous metastasis from a patient with distal esophageal/GEJ adenocarcinoma were utilized to generate the xenografts. De-identified patient samples were obtained with informed consent and xenografted as per Institutional Review Board (IRB)- and Institutional Animal Care and Use Committee (IACUC)-approved protocols (LAB-04-0979 and IACUC-00001501-RN01).

### Generation of subcutaneous PDX

Patient tumor was minced into ∼3-mm^3^ pieces within 10 min of removal from the patient. The minced tumor pieces were immersed in Matrigel (#356230, Corning, Bedford, MA, USA). Ectopic PDXs of GEJ cancer were generated by surgically implanting the tumor pieces into the flanks of six female (4 weeks old) SCID or athymic mice, as previously described (Rubio-Viqueira et al., 2006).

### Establishment of PDOX

Six-week-old female SCID mice were purchased from the Mouse Facility at our institute. For tumor implantation, the mice were anesthetized using 2% isoflurane and placed in the dorsal recumbent position with a heating pad beneath them. The mice were administered buprenorphine (0.05-0.10 mg/kg intraperitoneally immediately before surgery and postoperatively every 6-12 h for up to 72 h), and the fur over the ventral abdomen was clipped and disinfected (Fig. S1A). A ventral midline incision of ∼1.5 cm was made from the mid-abdominal region to the xiphoid process. The linea alba was incised similarly (Fig. S1B). The margins of the incisions were retracted, and the liver lobes were gently reflected with a moist cotton swab to allow visualization of the stomach and distal esophagus (Fig. S1C). The stomach was then lifted extracorporeally by placing traction on the greater curvature with forceps. A gavage needle was positioned underneath the distal esophagus to elevate it (Fig. S1D). Mechanical abrasion was then applied to the distal esophagus/GEJ with rat-tooth forceps (Fig. S1E), and a 1-mm^3^ minced tumor dipped in Matrigel was sutured to the esophagus/GEJ with a 6-0 polyglactin 910 suture (Fig. S1F). After implantation of the 1-mm^3^ PDX, the surgical site was observed for any bleeding. Once hemostasis was confirmed, the displaced organs were gently repositioned, and the linea was closed with a 4-0 absorbable suture. The skin was subsequently closed with wound clips (Fig. S1G). Ringer's lactate solution (1 ml) was administered subcutaneously to prevent dehydration (Fig. S1H). The mice were followed up daily, weighed two times per week and monitored for toxicity.

### MRI

Mice bearing PDOXs were anesthetized with 3% isoflurane for induction and ∼2% isoflurane for maintenance. Animals were placed onto the bed of a 7-T BioSpec MRI scanner with a 30-cm bore (Bruker BioSpin, Billerica, MA, USA). Two-dimensional (2D) respiratory-gated T2-weighted rapid acquisition with relaxation enhancement (RARE) images were acquired. Two-dimensional T1-weighted multi-slice multi-echo pre- and postcontrast images (Magnevist, Bayer, Whippany, NJ, USA) were acquired in the sagittal plane, and 2D T1-weighted fast-spoiled-gradient-echo pre- and postcontrast images were acquired in the axial plane.

### Radiation treatment to the PDOX

The PDOX-bearing mice were irradiated in a single 10-Gy fraction with a 250-kVp x-ray beam (15 mA, 0.35 mm copper filter) from a Philips RT 250 orthovoltage x-ray machine. An anesthetized mouse was placed inside a conforming cavity in a rubbery synthetic gel (Superflab) phantom placed on a 15-cm-thick acrylic block providing full backscatter. The field size was 5×5 cm, the source-to-skin distance was set at 50.0 cm, and a 5-mm-thick Wood's metal (Cerrobend) block with a 2.0×1.5-cm window was placed directly over the GEJ. The dose output in this irradiation geometry was 74.3 cGy/min (Fig. S2).

### Radiation response evaluation

RECIST version 1.1 was used to evaluate tumor response to therapy (Eisenhauer et al., 2009) Target lesions were chosen at baseline on the basis of modified RECIST criteria. These same target lesions were examined for the 2D volumetric analysis, which was conducted using ImageJ (Dello et al., 2007).

### Postmortem examination and histological analyses

After the mice were euthanized, the full extent of tumor growth was determined by autopsy. Gross examinations of the PDOX lesions and organs such as the lungs and liver were performed to assess the extent of locoregional spread. The PDOX tumors were measured using vernier calipers to determine their volume for comparison with the MRI volumetric measurements.

The tumors were measured *in situ* and excised, fixed with 4% formaldehyde, processed and embedded in paraffin blocks. Tumor sections (5 µm) were cut with a microtome (Microm HM355S; Thermo Fisher Scientific, Waltham, MA, USA). H&E, CD31 (#77699; CST, Denvers, MA, USA), Her2/neu (#790-2991; Roche, Santa Clara, CA, USA), p53 (PA0057; Leica Microsystems, Buffalo Grove, IL, USA), p16 (#705-4713; Roche, Santa Clara, CA, USA), EGFR (MAB13265; Abnova, Walnut, CA, USA) and CD68 (ab213363; Abcam, Cambridge, MA, USA) staining and validation were performed according to standard protocols in a Clinical Laboratory Improvement Amendments (CLIA)-designated laboratory following College of American Pathologists guidelines (Dello et al., 2007). A detailed antibody list can be found in Table S3. Slides were scanned at magnifications of 100× and 200×. Images of the slides were captured with a light microscope (ColorView I, BX43F; Olympus, Tokyo, Japan). A pathologist reviewed the stained sections to determine the histopathological features of the patient tumor and the xenograft tumors.

### Next-generation sequencing

A focus of tumor with higher than 70% tumor cellularity was marked on an H&E-stained section. Matching areas of the tumor were macrodissected for DNA sequencing. Normal squamous epithelium from the excised skin was used as a matched control. Briefly, we used a KAPA HyperPrep library preparation kit (Kapa Biosystems, Wilmington, MA, USA) to prepare indexed libraries from 200 ng genomic DNA that had been sheared using a Biorupter Ultrasonicator (Diagenode, Denville, NJ, USA). The multiplexed library pool was hybridized to the MD Anderson T200.1 probe pool (Roche NimbleGen, Madison, WI, USA). Following hybridization and reaction cleanup, the enriched libraries were amplified with seven cycles of postcapture PCR, then assessed for target enrichment by quantitative PCR. Sequencing was performed in one lane of a HiSeq4000 Sequencer (Illumina, San Diego, CA, USA) using a 150 bp paired-end configuration.

### Bioinformatic analysis

A customized hot spot somatic mutation bioinformatics pipeline focusing on analysis of 200 (T200) most commonly mutated genes was used. Mouse sequences were filtered out at the alignment. We aligned the T200 target-capture deep-sequencing data to human reference assembly hg19 using BWA (Li and Durbin, 2009) and removed duplicate reads using Picard (DePristo et al., 2011). We called SNVs and small indels using an in-house-developed analysis pipeline (Zhou et al., 2014) that classified variants into two categories – somatic and LOH – on the basis of variant allele frequencies in the tumor and matched normal tissues.

## Supplementary Material

Supplementary information
