# Supplementary material for: A novel patient-derived orthotopic xenograft model of esophageal adenocarcinoma provides a platform for translational discoveries
Source: Dis Model Mech. 2019 Dec 17;12(12):dmm041004. doi: 10.1242/dmm.041004 (PMC6918774; doi:10.1242/dmm.041004)
Supplement: Supplementary information [file dmm-12-041004-s1.pdf]

Supplemental Materials

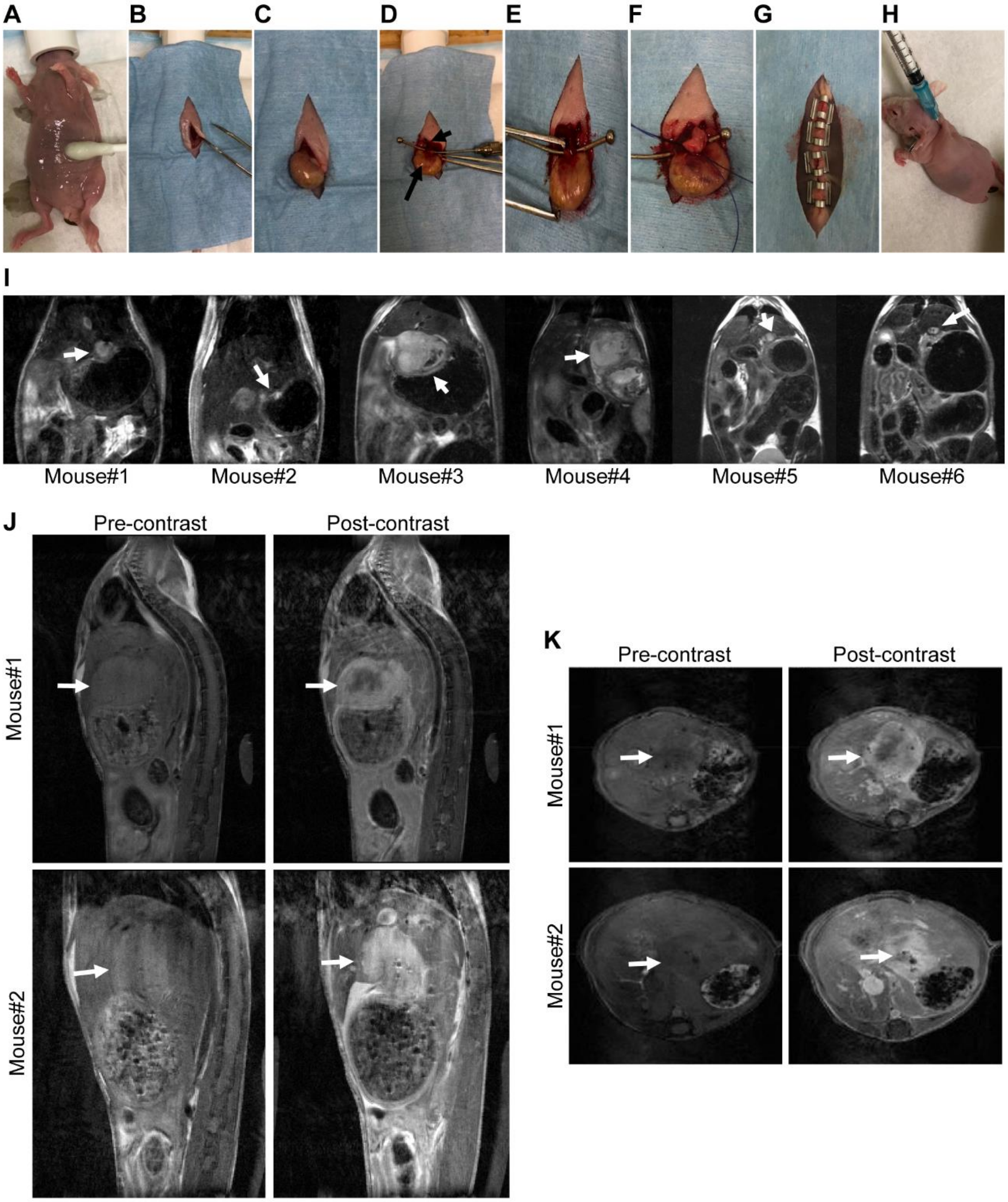

Figure S1. Surgical technique and tumor engraftment of the PDOX model. A, Surgical disinfection and scrub; B, Ventral midline incision of skin and musculature along the linea alba; C, Reflection of the lobes of liver to visualize the stomach; D, Elevation of the distal esophagus (small arrow) by passing a rigid oral gavage needle beneath it and retracting the stomach antrum for a better grip; E, Mechanical abrasion of the distal esophagus with rat-toothed forceps; F, Use of 6-0 absorbable suture to implant the tumor to the GEJ; G, Suturing of the musculature and closing of the incision with wound clips; H, Subcutaneous administration of Ringer’s lactate solution to prevent dehydration; I, T2 weighted MRI showing tumor engraftment in all 6 mice, with large tumors in mouse #3 and mouse #4; J and K, Pre- and postcontrast MRI, respectively, of PDOX showing tumor characteristics.

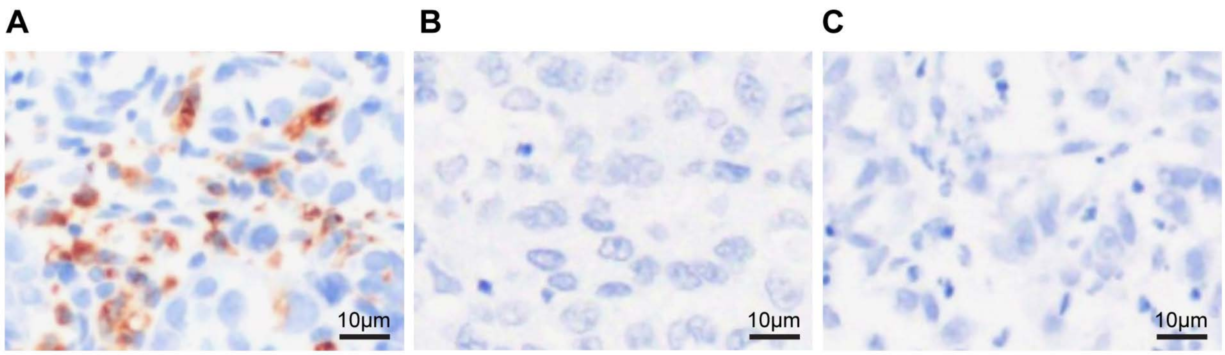

Figure S2. CD68 staining for human specific TAMS. A, patient tumor; B, subcutaneous PDX; C, PDOX. A, CD68+ve macrophages in patient tumor. B and C, CD68-ve macrophages in subcutaneous PDX and PDOX indicating macrophages of mouse origin. A-C, x200 magnification.

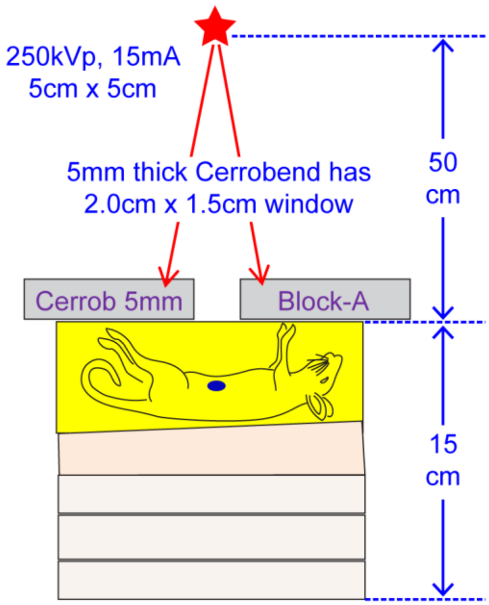

Figure S3. Schematic of the radiation treatment setup. The PDOX in the distal esophagus and GEJ was targeted using a Wood's metal (Cerrobend, Cerrob) block with a window placed directly over the GEJ. The red star indicates the radiation source.

Disease Models & Mechanisms • Supplementary information

Table S1. Genes showing loss of heterozygosity in patient`s tumor, subcutaneous PDX tumors, and PDOX tumors. HGNC_AAS, Official name of the gene with amino acid and protein position; Coverage, total number of DNA reads that cover the mutation site; Uploaded variation, genomic coordinates and nucleotide alteration; CDS position, position in the coding sequence; dbSNP, variant as per dbSNP database; genomes 1000 Allele Frequency, estimated percentage of mutant allele in Genomes 1000; Codons, alteration to codon; Consequence, translation consequence of the alteration; Condel, variant function predicted by Condel software; QUAL, Confidence score of a mutation (high if ≥50%, medium if 20-50%, and low if <20%); HGVSc, mutation transcript identifier based on HGVS convention.

Click here to Download Table S1

Table S2. Single-nucleotide variants representing somatic mutations in patient`s tumor, subcutaneous PDX tumors, and PDOX tumors. HGNC, Official name of the gene; Allele Freq, estimated percentage of mutant allele in the DNA sample; Coverage, total number of DNA reads that cover the mutation site; Uploaded variation, genomic coordinates and nucleotide alteration; CDS position, position in the coding sequence; Protein position, position in the protein product; Amino acids, alteration to amino acids; dbSNP, variant as per dbSNP database; Codons, alteration to codon; Consequence, translation consequence of the alteration; QUAL, Confidence score of a mutation (high if ≥50%, medium if 20-50%, and low if <20%); Location, genomic location; Allele, variant allele; Feature, the Ensembl transcript ID; cDNA position, position of the mutation in the transcript; HGVSc, mutation transcript identifier based on HGVS convention.

Click here to Download Table S2

Disease Models & Mechanisms • Supplementary information

Table S3. List of antibodies used for immunohistochemistry.

Biomarker	Vendor	Catalogue #	Clone	Antigen Retrieval	Dilution
**CD31 - Mouse**	Cell Signaling Technology	77699S	D8V9E	pH9, 20min at 100°C	1:100
**CD31 - Human**	Cell Signaling Technology	3528S	89C2	pH6, 20min at 100°C	1:100
**CD68**	Abcam	ab213363	EPR20545	pH6, 20min at 100°C	1:3000
**Her2/neu**	Roche	790-2991	4B5	pH6, 20min at 100°C	RTU 1:1
**P53**	Leica Microsystems	PA0057	DO-7	pH6, 20min at 100°C	RTU 1:1
**P16**	Roche	705-4713	E6H4	pH6, 20min at 100°C	RTU 1:1
**EGFR**	Abnova	MAB13265	31G7	pH6, 20min at 100°C	1:50
